# National Health Service consultants’ experiences and views of performance pay in England: a qualitative interview study

**DOI:** 10.1136/bmjopen-2025-111699

**Published:** 2026-09-28

**Authors:** Jo Parsons, Mark Exworthy, Nicholas Murphy, Julia Gauly

**Affiliations:** 1 Centre for Evidence and Implementation Science, University of Birmingham, Birmingham, UK; 2 Health Services Management Centre, University of Birmingham, Birmingham, UK; 3 Department of Immunology and Immunotherapy, University of Birmingham, Birmingham, UK

**Keywords:** QUALITATIVE RESEARCH, Health policy, Health Workforce, Hospitals

## Abstract

**Abstract:**

**Objectives:**

To explore National Health Service (NHS) consultants’ experiences and views of the local Clinical Excellence Award (CEA) scheme following changes to performance-related pay in England.

**Design:**

This was a qualitative study.

**Setting:**

This study was set in one NHS Trust in England.

**Participants:**

20 NHS consultants took part in the study. Out of these, seven had successfully applied for performance pay and 13 had not applied for performance pay in 2023 (one of these had applied unsuccessfully in a previous year). The majority of participants were male (n=11) and of White British ethnicity (n=12), and the majority had been qualified as a consultant for 10 or more years.

**Methods:**

Interviews were conducted online, audio-recorded, transcribed verbatim and analysed thematically using both inductive and deductive coding. Data collection continued until thematic saturation was reached. The reporting of this qualitative study was guided by the Consolidated Criteria for Reporting Qualitative Research (COREQ) 32-item checklist.

**Results:**

Five key themes were identified: (1) motivations for engagement with the scheme, (2) experience of the application process, (3) perceptions of fairness and equity and (4) influence of organisational and contextual factors. Participants described challenges with defining ‘excellence’, time pressures and discomfort with self-promotion. While the revised CEA structure was generally welcomed, concerns remained about inequities, the separation of research from clinical work and cultural barriers to sustainable service development.

**Conclusions:**

Consultants’ engagement with performance-related pay is shaped by personal, organisational and systemic factors. Although awards were valued as recognition and professional validation, performance pay was perceived to function more as symbolic recognition than as a motivator of performance. Findings highlight the need for transparent, equitable and team-oriented approaches to consultant recognition.

**STRENGTHS AND LIMITATIONS OF THIS STUDY**

- Data collection followed the Consolidated Criteria for Reporting Qualitative Research (COREQ) 32-item checklist, enhancing rigour, transparency and credibility.
- Semistructured interviews were conducted by trained, non-clinical researchers, reducing potential bias from peer influence.
- Purposive sampling sought to capture a range of perspectives, but voluntary participation resulted in a sample that was predominantly male, White British and established consultants, potentially under-representing groups most affected by inequalities in the award process.
- Data collection coincided with consultant industrial action in England in 2023, which may have influenced recruitment and participants’ perceptions of recognition.

## Introduction

 In general, performance-related pay (PRP)—where additional income for an employee is partially or fully linked to their performance^1^—is intended to motivate staff and improve performance outcomes.^2^ PRP is ‘a compensation scheme in which an employee’s pay is based partially or wholly on performance’.^1^ However, the use of PRP in healthcare organisations remains ‘contentious and inconclusive’.^3 4^ Concerns relate to the use of public money, the potential inequity between staff working in team-based settings and the problematic nature of measuring performance.^5–8^ Furthermore, there has been limited attention on PRP in relation to doctors, and especially their views of such schemes. While PRP can incentivise staff to work ‘harder’,^8^ the impact of monetary rewards on work effort may be limited, when doctors’ self-determination and intrinsic motivation are taken into account.^9^

A notable early example of performance pay in the UK healthcare system is the Distinction Awards Scheme for hospital consultants, introduced in 1948—the same year the National Health Service (NHS) was established.^10^ The scheme was a self-nomination process which rewarded senior doctors, dentists and academic general practitioners who made an outstanding contribution to delivering the goals of the NHS.^11^

In 2003, the Distinction Award Scheme was modified and renamed into Clinical Excellence Awards (CEAs) but largely remained a pay for performance scheme with consultants and academic general practitioners eligible to apply.^12^ The UK CEAs Scheme acknowledged and rewarded NHS consultants going above and beyond the typical expectations of their role.^13^ The scheme included 12 levels of awards, ranging from locally determined awards granted through a local process (Local Clinical Excellence Award, LCEA) to nationally evaluated bronze, silver, gold and platinum awards (National Clinical Excellence Awards).

Eligible candidates could apply through an online process, which was evaluated based on specific criteria designed to identify individuals who demonstrated a commitment to going ‘above and beyond’ their contractual duties.^13^ Successful applicants were awarded one of several levels, with the higher levels significantly increasing their overall salary. The average award amounted to approximately £12 800 per consultant, which was equivalent to around 11.8% of a consultant’s salary in 2016, highlighting the substantial financial impact of the award.^14^

The 2017 update to the 2003 contract made CEA awards contractual rather than optional, aiming to reform the awards process.^15 16^

After 2 years of the COVID-19 pandemic—during which CEA funding was evenly distributed among consultants in England, regardless of whether they had previously received an award, following an agreement between the government and the British Medical Association^14^—the National Clinical Impact Awards (NCIA) scheme was launched in 2022.^12 17^ The NCIA scheme separated local awards from national ones and placed responsibility for local awards under the authority of NHS Trusts.^12^

This meant that NHS Trusts were now responsible for deciding on the application domains, criteria and financial amounts for their performance pay schemes. However, under the consultant contract, CEA funding remained ring-fenced for consultants, meaning Trusts were not permitted to reallocate these funds to other staff groups or purposes.^18^

Despite multiple reforms over the years, the award schemes have been widely criticised for perpetuating persistent inequalities across gender, ethnicity, specialty and hospital type. Since the 1980s, studies have consistently shown that women, ethnic minority consultants, those in certain specialties and those working in non-teaching hospitals are less likely to receive awards.^12 17^ Reports such as *Mend the Gap*^19^ *a*nd *Surash Pearce*^20^ have highlighted how CEAs have contributed to the gender pay gap and disadvantaged ethnic minority consultants, particularly in the context of LCEAs—where one Trust reported a 24.5% lower average award value for ethnic minority consultants compared with their white peers.^20^

The application process has been criticised for its subjectivity, lack of transparency and continued emphasis on individual achievement in a team-based NHS.^12 14 21 22^ While the introduction of the NCIA scheme included increasing the number of awards, introducing improved guidance and enhancing data reporting,^23^ these reforms fall short of addressing core structural issues. There are no firm commitments to tackle systemic inequalities, and concerns remain about how criteria such as ‘impact’ will be fairly assessed across diverse specialties. The separation of NCIAs from local awards has raised further concerns, particularly around worsening transparency and deepening disparities. Additionally, the scheme continues to overlook the unique disadvantages faced by marginalised groups, including ethnic minority women and international medical graduates. Many critics view the reforms as largely cosmetic, with little evidence that the scheme improves patient care.

Despite its use of public funds, limited research has been conducted on performance pay schemes,^11^ particularly locally designed award schemes. This study examined the introduction of a newly designed PRP scheme in a single NHS Trust in England during 2022/2023. The implementation was accompanied by a comprehensive process of advertisement and engagement, which included multiple email communications and a series of workshops delivered both online and in person. Considerable time was spent defining and explaining what was expected from consultants in their applications, with all relevant information made available and widely publicised. Particular emphasis was placed on the difficulty of defining ‘excellence’, with ‘contribution’ highlighted instead as the primary criterion for assessment. Efforts were also made to encourage colleagues to share information directly with consultants who were harder to reach, to maximise awareness of the scheme.

Using a sequential explanatory mixed-methods design, we first conducted a survey to explore consultants’ experiences and views on performance pay and their experiences with the application process for the newly introduced local scheme; the findings from this survey have been published separately.^18^ Most consultants expressed support for CEAs in both principle and practice, although some were either neutral or unsupportive. Among those who did not apply in 2022, many cited doubts about their likelihood of success. CEAs were generally perceived as a form of professional recognition—particularly among women and part-time staff. However, views were mixed on whether part-time consultants should receive the full CEA payment.

Following the announcement of the award outcomes by the NHS Trust, we conducted semistructured interviews with consultants from the same Trust. We sought to capture a range of perspectives by inviting consultants who had successfully applied, unsuccessfully applied or chosen not to apply for the award. Recruitment emails and reminder emails explicitly emphasised our interest in hearing from all three groups. However, in this award round, all consultants who applied were successful, meaning there were no unsuccessful applicants available to participate. Consultants who chose not to apply did participate in the study, alongside the successful applicants. Participation was voluntary, and the recruitment period was also limited by the study timeline and funding constraints.

The aim was to explore consultants’ experiences and views of the newly introduced local performance pay awards system, including barriers to applying and experiences of the application process, and to examine how these experiences influenced their views on performance pay more generally.

As survey responses were collected anonymously, it was not possible to determine whether interview participants had also taken part in the survey. Consequently, survey and interview data could not be linked at individual level.

## Methods

The reporting of this qualitative study was guided by the Consolidated criteria for Reporting Qualitative research (COREQ) 32-item checklist (see online supplemental file 1).^24^

### Setting

This study was set in a Trust in England.

### Study design

We employed a qualitative study design using semistructured interviews with consultants on their views, experiences and perceived impact of performance pay.

### Recruitment and sampling

We sought to purposively recruit consultants with a range of experiences, including those who had received performance pay, those who had applied unsuccessfully and those who had not applied. We aimed to recruit between 15 and 25 consultants.

Once the decision about who had been awarded performance pay had been made by the trust, the administrator at the hospital site sent out information about the interview study to all consultants eligible for the award. Eligibility for the award required consultants to have been working at the site for at least 1 year, and only these consultants were contacted. A reminder email was sent out to all consultants to encourage participation in the study. Later, a reminder email was sent out specifically to those consultants who had unsuccessfully applied for performance pay. To preserve organisational confidentiality, the total number of NHS consultants approached is not disclosed. The Trust is a large acute care provider in England, operating across multiple sites and serving a diverse, predominantly urban population with high levels of deprivation. Its annual turnover exceeds £500 million.

Those who were interested in taking part then contacted the research team to find out more about the study. Those who expressed their interest, were then asked to indicate whether they applied for an award or not, and if they applied, whether they were given an award or not, and their specialty. The research team then invited participants to an online semistructured interview (conducted via MS Teams) based on the sampling frame.

Recruitment was completed in July 2023.

### Data collection

#### Positionality

Interviews were conducted by two non-clinical members of the research team—one female (JP) and one male (ME)—both trained health services researchers with experience in qualitative methods. Their non-clinical backgrounds meant they were not professional peers of the consultants, which may have reduced social desirability bias and encouraged open discussion. At the same time, this outsider status may have limited their ability to fully appreciate the nuances of medical culture and consultant career structures. Reflexivity was enhanced through regular team discussions, including with a coauthor who is an NHS consultant, to ensure interpretations remained grounded in clinical context and to minimise the influence of researcher assumptions on data analysis. Interviews were evenly split between the two researchers.

#### Interview process

Before the beginning of the interview, informed oral consent was obtained from all participants. Semistructured interviews took up to 60 min and followed a topic guide (see online supplemental file 2) that had been informed by the survey findings,^18^ existing literature and discussions between research team members and with stakeholders.

At the end of the interview, demographic information (years since qualification; gender; ethnicity; all in categories) was collected from participants using a digital form. This demographic information was requested to provide important context to the study data with regards to CEA disparity. Participants could choose ‘prefer not to say’ for any item.

Data collection continued until July 2023, at which point data saturation—defined in this study as the stage when no new themes emerged—was reached.^25^

### Data analysis

All interviews were recorded and transcribed professionally by a university-approved transcription service.

Thematic analysis was conducted by two research team members (ME and JP) to analyse the qualitative data, as this approach is well suited to identifying patterns and themes across qualitative data.^26^ Coding and analysis rarely operate in a purely inductive or deductive way; rather, most analyses involve a combination of both.^27^ In line with this, the qualitative data were analysed using both deductive and inductive approaches (ie, applying codes derived from the topic guide as well as developing additional codes from the data) to ensure that the themes were grounded in and closely linked to the data.^28 29^ Thematic analysis consisted of two processes: data management and data interpretation. Data interpretation consisted of familiarisation with the data through detailed multiple readings, coding the data (identifying phrases and assigning them a code which denotes their meaning), identifying patterns or themes among the coded data then reviewing the themes against the original data to ensure the conclusions drawn were supported by the original content of the interviews.

Codes were discussed by the research team and themes reviewed and defined iteratively. This collaborative and iterative process supported analytic rigour by enabling reflexive discussion of interpretations and ensuring consistency and coherence in coding and theme development across the dataset. Data saturation was achieved after the 17th interview. Three more interviews were conducted to confirm this. The themes and subthemes are presented in the form of text and a concept map. Quotes are used to substantiate analytic findings.

## Results

### Participants demographics

In total, 20 interviews were conducted (average length 45 min, ranging from 30 to 70 min). Although 23 consultants initially accepted the invitation, only 20 ultimately participated. Consultants are numbered 1–23, with numbers corresponding to those who accepted rather than those who participated. Out of the 20 interviews, 7 had successfully applied for performance pay and 13 had not applied for performance pay in 2023 (one of these had applied unsuccessfully in a previous year). The majority of participants were male (n=11) and of White British ethnicity (n=12), and the majority had been qualified as a consultant for 10 or more years. Table 1 shows aggregated participant characteristics.

**Table 1:** Participant characteristics

Characteristic	Number of participants
Gender	
Male	11
Female	9
Ethnicity	
White British	12
British Indian	1
Asian	4
British Pakistani	1
Black	1
Unknown	1
Sessions worked per week	
Fewer than 10	2
10 or more	17
Unknown	1
Years as a consultant	
10 years or less	4
More than 10 years	14
Unknown	1
Locum	1
Applied for award in 2023	
Yes (all applied in 2023)	7
Not 2023 but applied previously	8
Never applied	4
Unknown	1

### Experiences and views

Findings are categorised into five main themes: (1) motivations for engagement with the scheme, (2) experience of the application process, (3) perceptions of fairness and equity and (4) influence of organisational and contextual factors. The themes and subthemes identified in the analysis are presented in table 2 and visually summarised in a concept map (figure 1), providing an overview of the relationships between the key findings. The themes and subthemes are described in detail below and are illustrated with direct quotations from participants.

**Table 2:** Summary of themes and subthemes

Theme	Subthemes
Motivations for engagement with the scheme	Personal fulfilment and recognition as motivation to apply Organisational recognition as a motivation to apply Limited financial motivation Personality factors influencing likelihood of applying
Experience of the application process	Lack of visibility of the process Excellence not clearly defined Barriers to completing the application Views on the structure of the form
Perceptions of fairness and equity	Individual recognition for team effort Gaming the process Gender inequalities Equal distribution of awards during COVID-19
Influence of organisational and contextual factors	Reluctance to discuss money Imbalance between transparency and anonymity. Misaligned priorities

**Figure 1:**
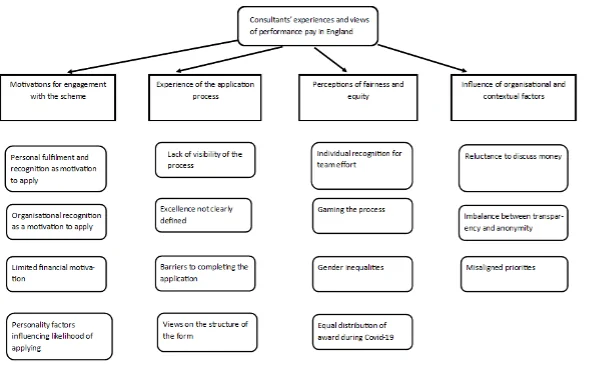
Concept map of themes and subthemes.

### Motivations for engagement with the scheme

Participants expressed a number of factors that influenced their decision to apply or not for performance pay as a consultant. These include personal, organisational, financial and personality factors influencing decisions of consultants.

#### Personal fulfilment and recognition as motivation to apply

Participants’ motivation to apply for a performance pay award was often influenced by the personal feelings such an award offered, such as self-worth, self-esteem and feelings of deserving the award. Going above and beyond was often reported as being done for personal job satisfaction, rather than being financially motivated.

… it’s nice to have the money, but actually I think a lot of it is about, somebody has looked at what I’ve done and said, “We think that’s valuable and we’re grateful that you’ve done it*.* (Consultant 11, female)

#### Organisational recognition as a motivation to apply

Organisational recognition (feeling appreciated by the organisation) was expressed by some participants. Feeling recognised or appreciated by the organisation or management was linked to increased motivation among the workforce, increased incentives to go above and beyond and increased staff retention.

I think the, the point about performance related pay for me is in part at least is about keeping people who are broadly doing the same job for 20 or 30 years, keeping, helping keeping them motivated or, or whatever in a role that … at least in some roles where you, you could just sit on your proverbial and just do the basics and perhaps be rewarded exactly the same as somebody who’s working hard*.* (Consultant 7, male)

#### Limited financial motivation

Many participants commented that the value of the award was insufficient to act as a motivating factor on its own. Additionally, participants reported concerns that receiving the award could have a negative impact on pensions, suggesting that financial elements alone were not motivating consultants to apply. This is further contextualised by the fact that consultants are able to earn additional income by working extra shifts or overtime as offered by the Trust, which may be a more flexible or preferable way to supplement earnings.

And the thought of putting the effort in to filling it in for an equivocal benefit. … ‘Cause I, if I got it this year, I wouldn’t get it next year, so you’d end up paying more tax this year and perhaps miss out next year. Actually when you look at them [CEAS] it’s not that great. (Consultant 6, male)

#### Personality factors influencing likelihood of applying

Participants reported that some personality traits were associated with applying for awards, with some types of individuals more or less likely to apply. Some individuals described not being confident enough to apply, with the process requiring certain personality traits, with reflections that individuals who are happier being vocal about their achievements will be more inclined to apply than others who are not confident about advertising their achievements. Feeling uncomfortable talking about own achievements was identified by some as a barrier to applying for an award.

I hate the process, I hate, I’m not someone who likes to sell myself. So, for me I find it an immensely stressful experience. And I’m one of those people who would always undersell myself rather than blow my own trumpet. … And so, I always feel that it is not suited to the people that are of my personality*.* (Consultant 9, female)

### Experience of the application process

Participants shared their experiences and awareness of the application process, and actual experience in filling out the form. For some the whole process felt shrouded in secrecy, while others struggled with defining what excellent performance looked like.

#### Lack of visibility of the process

Some participants reported a lack of visibility around the process of applying for an award, making elements of the process feel unknown and not publicly discussed. Some felt that the award processes generally were not talked about or advertised clearly, creating a feeling that people do not want to talk about it. The perception was that clearer or more visible promotion of the award would result in increased applications.

What I will say, is that I think, you know, the, if, if the reach of the advert is a lot more, I suspect that more people will probably apply. (Consultant 4. Female)

I think the way we do it is maybe a bit kind of secretive and undercover and people don’t really know much about it. (Consultant 1, Female)

#### Excellence not clearly defined

For some participants, it was difficult to define what excellence meant, especially without a clear or agreed way to measure it. Participants often reported feeling that excellence was subjective—its meaning varied depending on personal perspectives, professional roles, and contextual factors. Some also reflected on the limitations of existing criteria, noting that a consultant might be strong in some domains (such as teaching or service delivery) but less so in others. This raised concerns about how excellence was evaluated overall, and whether the current system fairly captured diverse contributions.

So, it’s about what you define as performance really, I think. That, for me, that, that was always the problem with the clinical excellence award because the people that were getting them were often not clinically excellent. They were very good at other bits of medicine, but they weren’t clinically in the building very much to be clinically excellent. (Consultant 13, Female)

#### Barriers to completing the application

Participants identified a number of barriers to the process of completing the application form. These included the time it takes to complete the form, and negative connotations of feeling like they were asking or begging for the award as a result of the self-nomination element required. These factors were identified as putting participants off applying.

I think most of the people that work the hardest don’t have the time to sit in their office and polish their form. (Consultant 6, male)

I think it’s just tiresome, I, I, I think, I think putting information on a form to try and get a little bit, it, it, it feels a bit like begging. You know. … I feel uncomfortable about pushing myself forward for money, you know, in a sort of slightly greedy, elbows out, pushing each other, people out the way. So, that’s not something I feel particularly comfortable doing. (Consultant 13, female)

#### Views on the structure of the form

Many participants reflected on the changes made to the scheme and the resulting changes to the application form (with the new style form offering a choice of which domains to complete), with many perceiving it as easier than previous versions, making the application process less of a barrier. The new approach was considered to be fairer, and allowed activities to be captured more appropriately.

and the old form with the five domains sort of encouraged people to try and have a, a, a breadth of, of input rather than necessarily being outstanding in one or two domains, ‘cause the way that the, the scheme worked is you, you couldn’t get, you might not get rewarded for that. So the, the newer system that we’ve had this year is a bit, it seems a bit more, it seems a bit better in that respect*.* (Consultant 7, male)

Despite positive reactions to the changes to the system and the application form, some limitations of the new system were reported. This included the separation of clinical and research elements, resulting in some applicants struggling to evidence research due to their clinical role.

But what happens there, then, is that people no longer have to, for example, tick a box for research. And certainly, when it comes then to deliver a standard national trial, it then means that the researchers are doing all of the work and that’s not spread out amongst the colleague. And what that’s then done, is that’s separated research from clinical rather than integrating it. And I think that’s been a, a step back and is a real challenge for any reconfiguration of the CEA system. (Consultant 19, male)

### Perceptions of fairness and equity

Elements of the process and the nature of the award were considered unfair by participants. This impacted on their desire and intention to apply for the award. This included perceptions around unequal opportunities for award-appropriate work, the nature of the awards not being able to recognise team input, some individuals signing up for quick-wins and unfair gender differences in opportunities for demonstrating excellence for the award.

#### Opportunities to go above and beyond

Participants highlighted concerns about how clinical performance is assessed and valued. Some consultants argued for a cross-specialty approach that rewards the best candidates overall. Some participants reported concerns that some specialties presented more opportunities to demonstrate going ‘above and beyond’, with some roles allowing more chance for research and publication, which others did not get time or opportunity to do these activities due to their specialism.

The more people seem to get rewarded for, for, for less time spent actually doing their employed job. And certainly… and also research, university employed, well, or honorary employed, so they, well, people who do research, I think if they got a, an NHS contract as well, they seem to benefit from lots of publications and things. Which is, you know, they’re paid by the university to do that. (Consultant 12, male)

#### Individual recognition for team effort

Some participants felt that performance pay awards were unfair as they recognise work of individuals while often the work has been conducted by the whole team. An individual award often felt unfair due to not recognising the contribution of the wider team.

It doesn’t take into account everybody else who facilitated me doing it. So, actually it should be a team recognition. Very rarely do, do, do we do things as absolute individuals although we may be the driving force*.* (Consultant 3, male)

Cause they’re assessing what you’ve done. So, I find that quite hard when it’s not just me. And it nearly always isn’t just me, ‘cause we, we’re teams. You know, we work in teams. And so, that’s quite horrible, you know, having to kind of write yourself up*.* (Consultant 23, female)

#### Gaming the process

Some participants felt that some individuals employed strategies when adopting tasks within their consultant role, favouring tasks that they felt would look good on the application, and would allow them to tick some boxes on the form and often not continuing with the role once the form had been submitted. Other participants felt that some people oversell the contribution of what they did to make it sound better. This was considered unfair by some participants.

So… …why not, why not do something amazing for, for one year. Write a pamphlet this year, great. You know. Because tick, tick, tick, I’ve written a patient pamphlet. Great, well done. You know. I’ve, I could run ten coffee mornings today, I could buy my own coffee, I could get my own room sorted out. I know that would count highly in the awards because above and beyond, self-driven, all those things and then I get, I get a, and then I don’t do them anymore. (Consultant 3, male)

So, one of the things that I realised very early on, it’s not what you’ve done, it’s how you express what you’ve done*.* (Consultant 4, male)

#### Gender inequalities

Participants described how women are often less likely to put themselves forward for awards or promotions compared with clinical awards due to systemic biases, lack of confidence and societal expectations. They noted that support from colleagues could make a critical difference, yet structural disadvantages remained, including the undervaluing of women’s work, assumptions about motivations and financial penalties linked to maternity leave. These barriers contribute to gender disparities in pay, such as fewer women applying for CEAs.

All of this stuff that you read in, in, in papers that, you know, women are less likely to apply and women perhaps of colour are even less likely to apply. Probably does apply to me, I mean if there was something clinical, if there was a clinical award going, I probably wouldn’t think twice of applying but we, we, with the, with the, with the performance pay I did actually*.* (Consultant 4, female)

Others emphasised that men’s work is often valued more highly and that women face disadvantages related to maternity leave, putting them at a perceived detriment when looking back over achievements in the period covered by the application form, if some or all of that time has been spent on maternity leave, leaving them with less opportunity to demonstrate excellence. This reinforces systemic inequities in recognition and reward.

And we know that the work of men is valued more highly than the work of women and we know that men are more employable than women…. And there’s this perception that women go to work ‘cause it’s fun, whereas men go to work to earn money to support a family, therefore their, their work should be paid more. (Consultant 11, female)

And I also think you’ve got to take into account that women go off and have babies and therefore their pregnancy or maternity leave shouldn’t be included in that time because certainly, I know, financially I’m much worse off than my male counterparts because I wasn’t there for two years, having my children and that doesn’t seem fair in this day and age that women are disenfranchised because of that, really. And, and as I say, I’d like to see that it’s, that’s an equitable split. I mean, I’m sure that there are significantly more men with clinical excellence awards in our organisation when you look at male to female ratios because of the way that the form has to be filled out, you know. So, they would be my thoughts really. (Consultant 13, female)

Together, these accounts illustrate how organisational and societal factors interact to limit women’s opportunities for professional advancement and equitable pay.

#### Equal distribution of awards during COVID-19

There were mixed views on the equalisation of awards during COVID-19. During the pandemic, rather than applications being made for performance pay awards, all eligible staff received an equal share. There were mixed feelings about whether the equalisation of awards during Covid, was appropriate or not. Many participants recognised the unique nature of the COVID-19 pandemic, and were accepting that under these circumstances, equalisation of the awards was an appropriate and fair way to do it. Participants acknowledged that people were not always working in their regular roles. However, the feeling was apparent that this approach was not appropriate outside of the unique pandemic environment.

I think it was appropriate during the pandemic cause we were all pitching in and helping in whatever format that was needed. And you know, people weren’t doing their normal day-to-day job. So I think it was very, very appropriate in the pandemic. I don’t think it would be appropriate to continue that because then people who are sitting back doing the minimum requirement would receive a bonus just for turning up and filling a seat. As opposed to people who are working really hard and going above and beyond getting kind of rewarded and recognised for that. (Consultant 1, female)

Other participants, on the other hand, spoke less favourably about the temporary move towards equalisation during the pandemic. Some felt a sense of unfairness that even those staff who were unable to work during the pandemic were entitled to a share of the payment, with no recognition for those working above their normal roles.

It felt like we went from one really deeply unfair system to another, because… …but unfair in a completely different way. We had a lot of people who worked incredibly hard, incredibly tough conditions and, and then for, for one reason or another, we had other people whose, who either couldn’t or wouldn’t or, and didn’t work in the same way. And yet everyone’s reward for it was the same. (Consultant 15, male)

### Influence of organisational and contextual factors

Existing factors, both within the organisation (including disparities highlighted both between the culture of the organisation and expectations on consultants and on the conflicting desire for transparency and anonymity) and wider contextual factors, were felt to influence the process, and whether consultants decided to apply for an award. There were conflicting views about whether the outcomes of the process should remain anonymous, with some consultants valuing anonymity while also wanting their achievements to be acknowledged.

#### Misaligned priorities

Some participants reflected on potential misalignment between work recognised by the award, and the culture of the organisation and expectations placed on consultants, which in turn shaped views about organisational structures and funding responsibilities. A recurring issue was the perceived link between CEAs and service development. For some, the award of a CEA for individual consultant-led initiatives was seen as potentially reducing the Trust’s impetus to formally develop or invest in those services. This perception may have created a disincentive to submit applications, as it raised concerns about whether CEAs were being used as a substitute for proper service planning and funding.

So, I disagree with the whole process. … Full stop. … Because, again, if, so, if somebody is, so, example. If I’m setting up a new service, and I'm not paid to do it, I think a new service is required. I work extra hours, I coordinate more things, I do it and I create a credible service for which I’m not getting currently paid. I want the organisation to decide whether this is a worthwhile thing to have, for me to have done. And if it’s worthwhile for me to have done, I’d prefer, then, I’d prefer the organisation to then fund the service that I’ve set up. Rather than giving me £3,000 to say, “There, there, well done. You still carry on doing it, we’re not going to fund it properly.” If it’s worthwhile doing, and you’re telling me it’s, I’ve done a good job, great. Fund the service. (Consultant 3, male)

#### Imbalance between transparency and anonymity

Many participants reflected on the difficult balance between awards remaining anonymous, and the desire for transparency of the process. This was discussed by participants, who often reflected that recognition is impossible if the process is anonymous—thus representing a complexity in feelings. For some, there was a difference between advertising award recipients and the financial amount they received internally among other staff, and externally to the public.

Because, as in, the, the current process doesn’t allow recognition by the organisation because it’s not a, because as I said, there’s no, it’s not transparent. I, my name and what I’ve done doesn’t appear on any publication so there’s no recognition and the PAs (Programmed Activities) meant to be anonymised. So, where’s the recognition? (Consultant 3, male)

But actually, if you had a transparent, you know, this person’s been awarded this award… … you know, for their work with XY or Z, a simple one line, that opens the floodgates for other people to get involved and to have transparency of sharing of information, not just transparency about the money. (Consultant 13, female)

#### Reluctance to talk about money

Many participants felt uneasy about talking about money with their colleagues, and therefore uneasy about others being aware that they had won an award. This reluctance to discuss finances led to a hesitation in having information about finances published for others to see, and in some cases this reluctance was considered a barrier to applying for an award.

I think a lot of people would be reluctant to have financial information about themselves published. Yeah I think it’s recognition of the award rather than the money*.* (Consultant 1, female)

People don’t like talking about money … … so I think it, it, it could, the fact, the very fact that people are hesitant about it and inevitably means that it probably would put some people off. (Consultant 7, male)

## Discussion

The study focused on consultants’ experiences and views of a newly introduced local performance pay awards system, including barriers to applying and experiences of the application process, and to examine how these experiences influenced their views on performance pay more generally. In total, 20 interviews with consultants were conducted in 2023 to explore consultants’ views and experiences of the local performance pay scheme at one NHS Trust in England.

Participants highlighted challenges in navigating the application process, including feelings of secrecy, difficulty defining ‘excellence’ and barriers such as time constraints and discomfort with self-promotion.

Participants generally welcomed the revised CEA application structure, finding it fairer and less burdensome, though some noted drawbacks such as the way the form separates research from clinical practice, which they felt risks undermining integration between the two. Concerns were also raised about the wider organisational culture, with CEAs sometimes viewed as being a substitute for proper service funding and investment rather than supporting sustainable service development. Motivations for applying were often driven more by personal satisfaction and organisational recognition than financial incentives, which were often seen as insufficient or potentially detrimental to pensions.

Participants perceived inequities in the process, including differences across specialties, individual rather than team recognition, gender-related barriers and ‘gaming’ of the system. Views on the equalisation of awards during COVID were mixed, with some seeing it as fair in exceptional circumstances and others viewing it as unfair. Anonymity in the process was seen as both protective and limiting, as it restricted recognition, while some participants expressed discomfort discussing monetary aspects of awards. Overall, the findings suggest a complex interplay of personal, organisational and systemic factors shaping engagement with performance pay.

In April 2024, consultants approved a new pay agreement,^30^ which incorporated LCEA budgets into basic pay.^18^ Nevertheless, the findings from this study offer important insights for the continued administration of NCIA. More broadly, the research enhances our understanding of the benefits, limitations and overall impact of PRP within the healthcare sector.

### Strengths and weaknesses of the study

This was the first qualitative study to explore consultants’ experiences and views on performance pay. The study adhered to the COREQ 32-item checklist, enhancing transparency and rigour in reporting qualitative research. Participants expressed both positive and negative views about the award process, including among those who had applied.

Purposive sampling was used to recruit consultants with a range of experiences of the award process (successful applicants, unsuccessful applicants and non-applicants), as well as a diversity of gender, ethnicity and career stage. Recruitment and reminder emails explicitly emphasised our interest in hearing from all of these groups. However, all consultants who applied in this award round were successful, meaning there were no unsuccessful applicants available to participate. In addition, participation was voluntary, and recruitment was constrained by the study timeline and available funding. Consequently, the final sample was predominantly male, White British, and established consultants, potentially under-representing the perspectives of women, consultants from minority ethnic backgrounds, and less established consultants, who may be disproportionately affected by inequalities in the award process. This may have limited the breadth of perspectives and experiences captured. A further limitation is that interviews focused exclusively on consultants and therefore did not capture the perspectives of other stakeholders, including patients, the public, managers or other staff. This is particularly relevant to transparency and accountability where awards are funded from public resources, as these stakeholders may have different views about the level of transparency that is appropriate or necessary.

In 2023, NHS consultants in England undertook significant industrial action—their first in nearly five decades—primarily in response to concerns over real-terms pay erosion and working conditions. The initial walkout occurred in July 2023. Protest driven by anger sends a powerful message, asserting a clear demand for recognition and value.^31^ We acknowledge that data collection coincided with this period of industrial action. This context may have significantly influenced both recruitment efforts—due to reduced availability and heightened workplace tensions—and consultants’ perceptions of recognition, potentially amplifying feelings of undervaluation or discontent.

### Comparisons with wider literature

Evidence from the public sector suggests that PRP does not necessarily improve motivation, satisfaction or performance.^32^ In healthcare, intrinsic motivations such as professionalism and commitment to patient care may be more important drivers of performance than financial rewards.^33^ This is consistent with literature suggesting that incentive-neutral approaches may better support clinicians’ intrinsic motivation than pay-for-performance schemes targeting specific areas of care, with professional values, reputation, feedback and opportunities for collaborative quality improvement identified as important.^8 34^ Our findings provide further insight into the limited motivational role of the financial component of CEAs. Participants did not generally view the financial value of awards as a strong motivator for participation, noting that additional income could often be matched or exceeded more easily through undertaking extra shifts than through completing the application process. This suggests that participation decisions were influenced by factors beyond financial reward, including the opportunity costs associated with applying. It also provides context for the separately reported survey findings that some consultants chose not to apply because of tax implications or lack of time.^18^ Instead, participants valued CEAs primarily for the recognition, professional esteem and self-worth they provided. This is relevant to the ‘crowding out’ literature, which suggests that external financial incentives can undermine intrinsic motivation when they are perceived as controlling or externally imposed.^35^ In contrast, CEAs may reinforce rather than crowd out intrinsic motivation. This interpretation is consistent with Exworthy *et al*, who found that CEAs could enhance intrinsic motivation because the scheme was professionally embedded, with doctors involved in its design and appraisal.^14^ The motivational effect of PRP may therefore depend not only on the financial reward itself, but on how it is designed, governed and interpreted within the professional context. This also helps explain the survey finding that PRP influenced consultants’ overall work commitment,^18^ with the interviews providing insight into the mechanisms underlying this relationship.

Gender inequalities in self-nomination and self-promotion provide an important context for understanding perceptions of fairness within performance and award schemes. Previous research has identified women’s lower propensity to self-promote or put themselves forward for awards as a barrier within self-nomination-based systems.^36^ Our interview findings are consistent with these observations, with participants describing women as potentially less likely to put themselves forward because of systemic biases, reduced confidence and wider societal expectations. These findings help to explain how apparently neutral award processes may reproduce gender inequalities. They also build on the survey findings, in which consultants perceived gender inequity in access to and success within the CEA scheme,^18^ by identifying some of the mechanisms that may underpin these perceptions.

The wider literature also highlights the potential for PRP and performance measurement schemes to generate unintended behavioural consequences, including misallocation of effort, tunnel vision and gaming, when individuals prioritise activities that are most easily measured or rewarded rather than those with the greatest organisational or societal value.^33 37^ In healthcare, Mannion and Braithwaite^38^ similarly identify unintended consequences of performance measurement, including measurement fixation, misplaced incentives, gaming and misrepresentation. Similar concerns emerged in our interviews, with consultants describing the selection of activities that would ‘look good’ on applications and the presentation or overselling of contributions to strengthen their claims for an award. These accounts raise questions about whether activities encouraged by the scheme necessarily correspond to those with the greatest impact on patient care or organisational performance. Rather than simply motivating greater effort, therefore, performance-based awards may influence which activities consultants choose to undertake, which contributions they prioritise and how they present those contributions.

The design of PRP schemes is particularly important in healthcare because performance is often dependent on teamwork, multidisciplinary collaboration and contributions that are difficult to attribute to individual staff members. Previous NHS approaches to linking pay to performance have focused largely on individual doctors and financial incentives, with less attention to team-based delivery, participative decision-making, quality of care and professional development.^39^ Wider evidence similarly suggests that individual-focused performance incentives can undermine teamwork and generate perceptions of unfairness in settings where outcomes depend on collaboration.^33^ Our interview findings reinforce these concerns, with some consultants questioning the fairness of awards that recognise individual rather than team contributions. The separately reported survey provides further context, showing opposition among some respondents to extending CEA funding to non-consultants.^18^ The 10-Year Health Plan for England’s proposal to extend PRP to individuals and teams across the NHS workforce therefore highlights the importance of carefully designed team-based approaches.^40 41^ Such approaches may provide an opportunity to address some of the limitations of individualised schemes, although their implementation will need to consider potential resistance among consultants and healthcare unions.^41^

Transparency and recognition represent a further tension within the design of award schemes. Although transparency is often regarded as important for accountability and perceptions of fairness, greater disclosure may also create concerns about privacy, financial sensitivity and public scrutiny. This tension was evident in our interviews. Participants recognised that transparency could make awards more meaningful by allowing colleagues to understand who had been recognised and why, and potentially encouraging others to contribute, but were more cautious about disclosure of financial amounts. These findings are consistent with the separately reported survey, in which respondents generally opposed full transparency but differed in their views about what information should be shared; around one-third supported sharing names or award levels with Trust peers, whereas many preferred financial details to remain private.^18^ Taken together, these findings suggest that, from the perspective of consultants in this study, transparency may be most acceptable when it supports recognition and accountability without requiring disclosure of sensitive financial information.

The interviews also highlighted uncertainty about the fairness and appropriate design of temporary changes to the award scheme during the COVID-19 pandemic. Participants’ accounts indicated that equalisation of awards raised broader questions about how equity should be operationalised within a performance-based system, particularly when exceptional circumstances affect opportunities to demonstrate individual performance. The separately reported survey similarly identified divergent views about whether temporary equalisation should be regarded as fair and whether it should continue.^18^ Although these findings reflect a specific response to the pandemic context, they also illustrate a broader challenge for performance-based schemes: the criteria used to define and reward individual performance may need to adapt when opportunities to achieve recognised outcomes are unevenly distributed. While the local CEA scheme has since closed, the national CEA scheme remains in place, meaning that the lessons from this study may have relevance for the ongoing design and implementation of CEA schemes at national level, particularly in considering how fairness and equity should be addressed when circumstances affect opportunities to demonstrate individual performance.

Overall, the interview findings suggest that the CEA scheme operates through a complex combination of professional recognition, identity, perceived fairness and behavioural incentives rather than through financial motivation alone. Participants’ accounts indicate that individualised performance awards can reinforce professional esteem and commitment, but may also reproduce inequalities, encourage strategic behaviour and undervalue collaborative contributions.

### Implications for policy and practice

This study highlights the complex and often conflicting views consultants hold about PRP and the CEA scheme. While such awards can provide recognition, personal validation and enhance professional identity, structural and cultural barriers undermine their fairness and effectiveness. Survey and interview findings indicate persistent gender inequities, inequities across specialties and advantages for those more comfortable with self-promotion, potentially disadvantaging consultants whose contributions are substantial but less visible. Some participants described strategically selecting tasks that ‘tick the box’ on applications, often discontinuing them afterwards, raising questions about whether current systems incentivise sustained excellence. This concern is consistent with evidence from the UK Quality and Outcomes Framework, where financial incentives improved recorded quality of care in the short term but showed less consistent benefits over longer periods, with gains often reversing after incentives were withdrawn.^42^ Similarly, evaluation of the NHS Prescribed Specialised Services Commissioning for Quality and Innovation programme found that most pay-for-performance schemes had little or no measurable impact on quality improvements in specialised services, suggesting that incentives alone may be insufficient to drive sustained change when targets are complex or focused on specific measurable outcomes.^43^ A wider review across Organisation for Economic Co-operation and Development countries also found mixed effects, with only just over half of studies demonstrating improved healthcare worker performance, emphasising the importance of carefully designed incentives that promote meaningful outcomes rather than narrow target achievement.^44^ These findings suggest that PRP may be more effective when tied to long-term, demonstrable impact rather than short-term, easily measurable activities. Policymakers and employers should therefore consider revising award criteria to place greater emphasis on sustained improvements in patient care, service development, education, research and leadership over time, rather than isolated achievements.

Financial incentives alone were found to have limited influence on participation, with intrinsic motivators such as professionalism, patient-centred improvement and team contributions playing a stronger role. Individual-focused PRP schemes may also undermine collaboration, as current awards predominantly recognise individual rather than team contributions. With the 10-Year Health Plan for England proposing the expansion of financial rewards to high-performing teams, leaders and organisations, policymakers should pilot and rigorously evaluate team-based reward models before wider implementation, assessing their effects on collaboration, staff morale, equity and patient outcomes. Employers can support these initiatives by embedding team-based recognition within appraisal systems, ensuring that contributions from multidisciplinary teams are acknowledged alongside individual achievement.

The study also revealed tensions between recognition and privacy. Consultants valued visibility of achievements to inspire peers and improve organisational culture but were cautious about disclosing financial details. Policymakers should therefore develop transparent but proportionate award processes, clearly communicating the criteria and rationale for awards while protecting individual financial confidentiality. Ensuring that professionals understand how decisions are made and feel involved in the process is essential to translating financial recognition into genuine engagement rather than perceptions of unfairness or favouritism.^45^ Employers should complement these reforms by providing structured mentoring, application support and leadership development opportunities for under-represented groups, alongside regular equality audits to identify and address disparities in award applications and outcomes across gender, specialty and other protected characteristics. Clearer and more inclusive definitions of excellence, supported by assessor training to minimise bias, may further improve confidence in the fairness of the process.

In summary, PRP in healthcare appears to function more as symbolic recognition than as a driver of measurable performance. To maximise its effectiveness, policymakers should design reward systems that prioritise sustained impact, collaboration and equity, while employers should ensure that implementation is transparent, inclusive and supported by robust monitoring and evaluation. Together, these measures could help ensure that PRP promotes meaningful improvements in workforce motivation, organisational culture and ultimately, patient care.

### Unanswered questions and future research

While this study provides valuable insights, several important questions remain. Further research is needed to explore how PRP schemes could be restructured to recognise both individual and team contributions more equitably, and to understand their long-term impact on workforce motivation, morale and retention. Evidence on the effects of PRP for lower-earning NHS employees, such as nurses, is currently limited,^33^ representing a key gap. Future studies should also investigate barriers faced by women and consultants from minority ethnic backgrounds to identify targeted interventions that reduce disparities in applications and awards. Comparative research across different NHS Trusts or health systems could clarify how local organisational cultures influence engagement with performance pay. Finally, there is scope to evaluate alternative models of recognition beyond financial incentives, to determine which approaches most effectively support sustained excellence, collaboration and workforce well-being in clinical practice.

## Conclusions

This study is the first qualitative investigation into consultants’ views and experiences of PRP within the NHS, providing unique insights into the personal, organisational and systemic factors shaping engagement with the CEA scheme. Our findings highlight that while consultants value recognition and professional validation, structural barriers, perceived inequities and cultural norms—particularly around gender, self-promotion and team versus individual recognition—affect participation and perceptions of fairness. Financial incentives alone were not primary motivators; intrinsic factors such as professional satisfaction, organisational acknowledgement and patient-centred impact played a stronger role. These insights suggest that performance pay functions more as symbolic recognition than as a driver of measurable performance and underscore the need for policies that promote equity, transparency and team-oriented recognition while balancing privacy and organisational culture. Future research should examine the long-term impact of evolving PRP models, particularly team-based reward systems, on workforce motivation, collaboration, retention and patient outcomes. Longitudinal and multisite studies are also needed to evaluate whether policy changes reduce inequalities in award access and outcomes, and to identify strategies that support fairer, more inclusive recognition of excellence across the consultant workforce. Such evidence will be essential to inform the development of reward systems that not only recognise excellence but also foster sustained improvements in organisational performance and patient care.

## Supplementary material

10.1136/bmjopen-2025-111699 online supplemental file 1

10.1136/bmjopen-2025-111699 online supplemental file 2

## Data Availability

All the data are available upon reasonable request.

## References

[1] Abner GB, Kim SY, Perry JL (2017). Building Evidence for Public Human Resource Management: Using Middle Range Theory to Link Theory and Data. *Review of Public Personnel Administration*.

[2] Bloor K, Maynard A (2012). Reform of the clinical excellence awards scheme: why are we waiting?. J R Soc Med.

[3] Ahmad F, Lyu J (Daisy, Stazyk E (2024). Back to the future revisited: A systematic literature review of performance-related pay in the public sector. International Public Management Journal.

[4] Perry JL, Engbers TA, Jun SY (2017). Back to the Future? Performance-Related Pay, Empirical Research, and the Perils of Persistence.

[5] Kessler I, Purcell J (1992). Performance related pay: Objectives and application. *Human Res Mgmt Journal*.

[6] Dowling B, Richardson R (1997). Evaluating performance-related pay for managers in the National Health Service. *The International Journal of Human Resource Management*.

[7] Carter N, Day P, Klein R (2002). How Organisations Measure Success. How Organisations Measure Success.

[8] Bellé N (2015). Performance‐Related Pay and the Crowding Out of Motivation in the Public Sector: A Randomized Field Experiment. Public Adm Rev.

[9] Deci EL, Ryan RM (2000). The “What” and “Why” of Goal Pursuits: Human Needs and the Self-Determination of Behavior. Psychol Inq.

[10] Young GJ, Beckman H, Baker E (2012). Financial incentives, professional values and performance: A study of pay‐for‐performance in a professional organization. J Organ Behavior.

[11] Treadgold BM, Campbell JL, Abel GA (2023). Investigating Clinical Excellence and Impact Awards (INCEA): a qualitative study into how current assessors and other key stakeholders define and score excellence. BMJ Open.

[12] Essex R, Rao M, Mir F (2022). The National Clinical Impact Awards: cosmetic change or fundamental reform?. J R Soc Med.

[13] Campbell JL, Abel G, Campbell J (2016). Clinical excellence: evidence on the assessment of senior doctors’ applications to the UK Advisory Committee on Clinical Excellence Awards. Analysis of complete national data set. *BMJ Open*.

[14] Exworthy M, Hyde P, Mcdonald-kuhne P (2016). Knights and Knaves in the English Medical Profession: the Case of Clinical Excellence Awards. J Soc Pol.

[15] BMA; NHS Employers (2018). Local clinical excellence awards guidance 2018-2021.

[16] Pay and conditions circular (M&D) 1/2018 changes to terms and conditions of service and model contract for consultants: clinical excellence awards.

[17] Esmail A, Abel P, Everington S (2003). Discrimination in the discretionary points award scheme: comparison of white with non-white consultants and men with women. BMJ.

[18] Exworthy M, Gauly J, Parsons J (2025). Performance-related pay for NHS consultants: exploring views and perceived impacts in one NHS Trust in England. *BMJ Lead*.

[19] Dacre J, Woodhams C, Atkinson C (2020). Mend the Gap: The Independent Review into Gender Pay Gaps in Medicine in England.

[20] (2019). The surash pearce report a comprehensive review into ethnic pay-gap and workforce development at the newcastle upon tyne hospitals nhs foundation trust.

[21] Essex R, Talagala I, Dada O (2021). Clinical Excellence Awards-time for a fairer NHS rewards scheme. BMJ.

[22] (2005). Performance-Related Pay Policies for Government Employees.

[23] Reforming the national clinical excellence awards scheme: response from dhsc and welsh government GOV.UK. https://www.gov.uk/government/consultations/reforming-the-national-clinical-excellence-awards-scheme/outcome/reforming-the-national-clinical-excellence-awards-scheme-response-from-dhsc-and-welsh-government.

[24] Tong A, Sainsbury P, Craig J (2007). Consolidated criteria for reporting qualitative research (COREQ): a 32-item checklist for interviews and focus groups. Int J Qual Health Care.

[25] Qualitative and action research: a practitioner handbook - michael p. grady - google books. https://books.google.co.uk/books?hl=en&lr=&id=JOr3-A3-LbwC&oi=fnd&pg=PA1&dq=Grady,+M.+P.+(1998).+Qualitative+and+action+research:+A+practitioner+handbook.+Phi+Delta+Kappa+International.&ots=hDXQWcpBM_&sig=unCXvQHguCUj-FnbArK2Gbclnh4#v=onepage&q&f=false.

[26] Alhojailan MI (2012). Thematic analysis: A critical review of its process and evaluation. West East Journal of Social Sciences.

[27] Byrne D (2022). A worked example of Braun and Clarke’s approach to reflexive thematic analysis. Qual Quant.

[28] Patton MQ (2014). Qualitative Research & Evaluation Methods: Integrating Theory and Practice.

[29] Fereday J, Muir-Cochrane E (2006). Demonstrating Rigor Using Thematic Analysis: A Hybrid Approach of Inductive and Deductive Coding and Theme Development. Int J Qual Methods.

[30] Murphy N, Exworthy M, Gauly J (2024). The end of local clinical excellence awards. BMJ.

[31] Ramsey SM (2025). Diagnosis: undervalued. A psychoanalytic exploration of doctor strikes in the British National Health Service, 2023-2024. Med Humanit.

[32] Bryson A, John M Title: how much performance pay is there in the public sector and what are its effects? running title: performance pay in the public sector.

[33] Ray K, Foley B, Tsang T (2014). A review of the evidence on the impact, effectiveness and value for money of performance-related pay in the public sector.

[34] Berenson RA, Rice T (2015). Beyond Measurement and Reward: Methods of Motivating Quality Improvement and Accountability. Health Serv Res.

[35] Frey BS, Jegen R (2001). Motivation Crowding Theory. J Econ Surv.

[36] Women Don’t Self-Promote, But Maybe They Should - Professional &amp; Executive Development | Harvard DCE. https://professional.dce.harvard.edu/blog/women-dont-self-promote-but-maybe-they-should/#The-Gender-Promotion-Gap.

[37] Bevan G, Hood C (2006). WHAT’S MEASURED IS WHAT MATTERS: TARGETS AND GAMING IN THE ENGLISH PUBLIC HEALTH CARE SYSTEM.

[38] Mannion R, Braithwaite J (2012). Unintended consequences of performance measurement in healthcare: 20 salutary lessons from the English National Health Service. Intern Med J.

[39] Buchan J, Seccombe I, Gershlick B (2017). In short supply: pay policy and nurse numbers workforce profile and trends in the english NHS.

[40] Department of Health and Social Care (2025). Fit for the future: 10 year health plan for England.

[41] Exclusive: performance-related pay proposed in 10-year plan | news | health service journal. https://www.hsj.co.uk/workforce/exclusive-performance-related-pay-proposed-in-10-year-plan/7039549.article.

[42] Ho L, Mercer SW, Henderson D Effect of UK Quality and Outcomes Framework pay-for-performance programme on quality of primary care: systematic review with quantitative synthesis. *BMJ*.

[43] Feng Y, Kristensen SR, Lorgelly P (2024). Pay-for-Performance incentives for specialised services in England: a mixed methods evaluation. *Eur J Health Econ*.

[44] Lin TK, Werner K, Witter S (2022). Individual performance-based incentives for health care workers in Organisation for Economic Co-operation and Development member countries: a systematic literature review. *Health Policy*.

[45] Lmc S Effects of Pay for Performance on Motivation and Professional.

