# Supplementary material for: National Health Service consultants’ experiences and views of performance pay in England: a qualitative interview study
Source: BMJ Open. 2026 Sep 28;16(9):e111699. doi: 10.1136/bmjopen-2025-111699 (PMC13636320; doi:10.1136/bmjopen-2025-111699)
Supplement: Supplementary file 1 [file bmjopen-2025-111699.reviewer_comments.pdf]

PEER REVIEW HISTORY

BMJ Open publishes all reviews undertaken for accepted manuscripts. Reviewers are asked to complete a checklist review form and are provided with free text boxes to elaborate on their assessment. These free text comments are reproduced below.

ARTICLE DETAILS

Title (Provisional)

National Health Service consultants' experiences and views of performance pay in England: A qualitative interview study

Authors

Parsons, Jo; Exworthy, Mark; Murphy, Nicholas; Gauly, Julia

VERSION 1 - REVIEW

**Reviewer**	**1**
**Name**	**Stringer, Gemma**
**Affiliation**	**University of Manchester, Division of Population Health**
**Date**	**30-Mar-2026**
**COI**	**None**

This paper explores an important and under researched topic. The study is timely and relevant, and it offers useful insights into how recognition and fairness of the clinical excellence awards scheme affects consultants' engagement with it. The qualitative approach is appropriate, and the findings have the potential to inform policy and practice.

However, several areas need further development, particularly the depth and clarity of the analysis, the presentation of the findings, and the explanation of how the survey and interview components relate to each other.

Major Comments

The findings rely too heavily on multiple quotes, sometimes with limited explanation of what they show. The analysis needs to be clearer and more developed. I recommend reducing the number of quotes in places, adding more interpretation around each one, and ensuring each sub-theme is well defined and distinct. Some sub-themes e.g. organisational culture, definitions of excellence seem broader than the theme they are under and may need moving.

Several parts of the paper imply that the survey findings are reported here, which is confusing. Please make it clear that the survey is published separately. In the discussion, limit comparison with the survey to one focused paragraph, and use the rest of the discussion to engage with the wider literature. As written, the discussion sometimes reads as a comparison between two parts of the same study.

More information is needed about attempts to recruit unsuccessful applicants, why this was not possible, and how data saturation was judged without their perspectives. Since the award is described as perpetuating inequalities, the limitations should also acknowledge the lack of diversity in the sample.

Specific comments by page

Page 4

Lines 10-11: Acknowledge lack of diversity in the limitations.

Line 45: 'yardstick' may be too colloquial.

Lines 49-59: Clarify that the survey is published separately.

Page 5

Lines 3-7: Reword to avoid implying unsuccessful candidates were interviewed - expand on recruitment attempts.

Lines 8-10: Reference to the survey is confusing - remove or clarify.

Lines 11-17: Paragraph feels misplaced - consider moving to the discussion.

Line 23: Missing word; "consolidated" should be lowercase.

Line 52: Wording unclear.

Line 60: Explain why only consultants with one year on site could be contacted.

Page 7

Lines 8-11: The justification for TA over other methods is overstated. Other methods e.g. grounded theory, IPA, discourse analysis all involve interpretative analysis. I recommend revising the sentence to say TA is suitable or well-established.

Line 12: According the Braun and Clarke codes do not 'arise'. I suggest revising the wording. See Byrne, D. A worked example of Braun and Clarke's approach to reflexive thematic analysis. Qual Quant 56, 1391–1412 (2022).

Page 8

Some sub-themes appear misplaced or too broad - consider repositioning (see major comment).

Line 56: 'Given pandemic' needs rephrasing.

'Equalisation' requires further explanation.

Page 9

Line 55: Should read 'begging for the award.'

Page 10

Line 38: Expand analysis so the quote is not doing all the work.

Line 49: Organisation culture seems quite broad here, this seems to be about misaligned incentives. If it's about culture I think this needs expanding and moving into a different section.

Page 11

Line 37: Missing space.

Page 13

Lines 4-9: Quote needs clearer contextualisation (specialties, research time).

Lines 12-35: Three similar quotes - consider reducing and strengthening analysis.

Lines 42-52: Expand on how the form is completed and how this disadvantages women returning from maternity leave.

Page 15

Line 37: Section contains quotes with minimal analysis - I suggest expanding the narrative.

Overall: Too many quotes in succession. I suggest developing the interpretive commentary.

Page 16

Final theme also relies heavily on quotes – I suggest strengthening the analysis here and do not leave the quotes to do the heavy lifting.

Page 17

Lines 44-46: I would have thought that non-clinical researchers in health research are fairly common is this commonly cited as a strength?

Many strengths listed are standard qualitative practice. I suggest focusing on what is distinctive.

Line 58: Provide more detail on why unsuccessful applicants could not be recruited.

Page 18

Lines 33, 51, 57: Clarify that survey findings are from a separate publication and connect them more explicitly to the interview findings. I suggest stating the findings from the survey more clearly as in places it relies on the reader checking the other publication.

Reviewer **2**

Name **Nowrouzi-Kia, Behdin**

Affiliation **University of Toronto, Occupational Science and Occupational Therapy**

Date **03-Jun-2026**

COI **None**

Thank you for the opportunity to review your work. This study from the United Kingdom explores the National Health Service consultants perspective and lived-experience of the local clinical excellence award. Overall, it is a noteworthy study that provides insight on consultants views on the changes to performance-related pay in England. However, the paper has issues that must be be addressed before it may be accepted for publication. I have

structured my feedback for each section of the paper. I'd be happy to review the paper again, after the authors have had the opportunity to review, revise and resubmit their work.

Abstract

The authors should include the use of the COREQ checklist in this section. Moreover, please sell out the NHS in the abstract. Given the international audience of the journal, this is important to improve the readers understand of the context of the study.

Introduction

This is a well written section that highlights the history of the NHS and the CEA. I'd like to see a stronger connection with the literature on the topic and the rationale for this study. The knowledge gap that the study is attempting to address needs to be further strengthened and articulated in the section. For the benefit of the reader, please provide a clear objective/aims/purpose statement.

Methods

The authors have included the COREQ checklist. For the data analysis section, the authors need to expand the use of the thematic analysis, why they used both an decutive and inductive approach as well as an expanded discussion of the methodological rigor.

Resutls

It would be helpful if the authors also provided a concept map of the study themes and subtheme in additional to table 2. This will be a helpful visual to provide a high-yield overview of the study findings.

Discussion

The structure of this section should be improved. I would suggest restating the purpose of the study, expanding on the results section using a critical lens and aligning the findings with the extant literature. I would also suggest expanding and delving into providing concrete recommendations for policy makers and employers. The limitation and conclusion sections should be expanded, including more details on future research consideration

---

VERSION 1 - AUTHOR RESPONSE

**Reviewer: 1 - Dr. Gemma Stringer, University of Manchester**
**Comments to the Author:**<br>**This paper explores an important and under researched topic. The study is timely and relevant, and it offers useful insights into how recognition and fairness of the clinical excellence awards scheme affects consultants' engagement with it. The qualitative approach is appropriate, and the findings have the potential to inform policy and practice.**<br>**However, several areas need further development, particularly the depth and clarity of the analysis, the presentation of the findings, and the explanation of how the survey and interview components relate to each other.**
Many thanks for taking your time to review our manuscript and for all your valuable and detailed comments. We have addressed all of them and feel that they have helped to substantially improve our manuscript.
**Major Comments**

The findings rely too heavily on multiple quotes, sometimes with limited explanation of what they show. The analysis needs to be clearer and more developed. I recommend reducing the number of quotes in places, adding more interpretation around each one, and ensuring each sub-theme is well defined and distinct. Some sub-themes e.g. organisational culture, definitions of excellence seem broader than the theme they are under and may need moving.

Several parts of the paper imply that the survey findings are reported here, which is confusing. Please make it clear that the survey is published separately. In the discussion, limit comparison with the survey to one focused paragraph, and use the rest of the discussion to engage with the wider literature. As written, the discussion sometimes reads as a comparison between two parts of the same study.<br>More information is needed about attempts to recruit unsuccessful applicants, why this was not possible, and how data saturation was judged without their perspectives. Since the award is described as perpetuating inequalities, the limitations should also acknowledge the lack of diversity in the sample.
Many thanks for your very detailed and helpful comments. We have addressed all of your major comments as outlined below.
**Specific comments by page**
**Page 4, Lines 10-11: Acknowledge lack of diversity in the limitations.**
We have revised the third bullet point to acknowledge the lack of diversity in the 'strength and limitations' section:<br>• "Purposive sampling sought to capture a range of perspectives, but voluntary participation resulted in a sample that was predominantly male, White British, and established consultants, potentially underrepresenting groups most affected by inequalities in the award process."
**Page 4, Line 45: 'yardstick' may be too colloquial.**
Thank you - we have replaced the term 'yardstick' now:<br>Particular emphasis was placed on the difficulty of defining "excellence," with "contribution" highlighted instead as the **primary criterion** for assessment.
**Page 4, Lines 49-59: Clarify that the survey is published separately**
We have clarified that the survey is published separately:<br>"Using a sequential explanatory mixed-methods design, we first conducted a survey to explore consultants' views on performance pay and their experiences with the application process for the newly introduced local scheme; the findings from this survey have been published separately (11)."
**Page 5, Lines 3-7: Reword to avoid implying unsuccessful candidates were interviewed - expand on recruitment attempts.**
We have reworded this section to avoid implying that unsuccessful candidates were interviewed and have expanded the description of the recruitment attempts:<br>"Following the announcement of the award outcomes by the NHS Trust, we conducted semi-structured interviews with consultants from the same Trust. We sought to capture a range of perspectives by inviting consultants who had successfully applied, unsuccessfully applied, or chosen not to apply for the award. Recruitment emails and reminder emails explicitly emphasised our interest in hearing from all three groups. However, in this award round, all consultants who applied were successful, meaning there were no unsuccessful applicants available to participate. Participation was voluntary, and the recruitment period was also limited by the study timeline and funding constraints."

We have also updated the wording in the limitations section:<br>"Purposive sampling was used to recruit consultants with a range of experiences of the award process (successful applicants, unsuccessful applicants, and non-applicants), as well as a diversity of gender, ethnicity, and career stage. Recruitment and reminder emails explicitly emphasised our interest in hearing from all of these groups. However, all consultants who applied in this award round were successful, meaning there were no unsuccessful applicants available to participate. In addition, participation was voluntary, and recruitment was constrained by the study timeline and available funding. Consequently, the final sample was predominantly male, White British, and established consultants, potentially underrepresenting the perspectives of women, consultants from minority ethnic backgrounds, and less established consultants, who may be

disproportionately affected by inequalities in the award process. This may have limited the breadth of perspectives and experiences captured."
**Page 5, Lines 8-10: Reference to the survey is confusing - remove or clarify.**
Thank you for this comment. We have clarified rather than removed this statement because, in a sequential explanatory mixed-methods design, readers may expect the survey and interview data to be linked. We therefore felt it was important to explain that survey responses were anonymous, making it impossible to determine whether interview participants had also completed the survey and preventing individual-level linkage of the datasets. We have reworded line 8-10 as follows:<br>"As survey responses were collected anonymously, it was not possible to determine whether interview participants had also taken part in the survey. Consequently, survey and interview data could not be linked at the individual level."
**Page 5, Lines 11-17: Paragraph feels misplaced - consider moving to the discussion. Major comment on discussion: In the discussion, limit comparison with the survey to one focused paragraph, and use the rest of the discussion to engage with the wider literature. As written, the discussion sometimes reads as a comparison between two parts of the same study.**
Thank you for this valuable suggestion. In response, we have moved this paragraph to the Discussion section (see the final paragraph before the section entitled "Strengths and Weaknesses of the Study") and have substantially revised the Discussion to provide greater engagement with the wider literature. All changes are highlighted in the revised Discussion section. For example, we have added the following text, incorporating recent references:<br>"This concern is consistent with evidence from the UK Quality and Outcomes Framework, where financial incentives improved recorded quality of care in the short term but showed less consistent benefits over longer periods, with gains often reversing after incentives were withdrawn (41). Similarly, evaluation of the NHS Prescribed Specialised Services Commissioning for Quality and Innovation (PSS CQUIN) programme found that most pay-for-performance schemes had little or no measurable impact on quality improvements in specialised services, suggesting that incentives alone may be insufficient to drive sustained change when targets are complex or focused on specific measurable outcomes (42). A wider review across Organisation for Economic Co-operation and Development (OECD) countries also found mixed effects, with only just over half of studies demonstrating improved healthcare worker performance, emphasising the importance of carefully designed incentives that promote meaningful outcomes rather than narrow target achievement (43)."<br>(…) "Ensuring that professionals understand how decisions are made and feel involved in the process is essential to translating financial recognition into genuine engagement rather than perceptions of unfairness or favouritism (44)."

**Page 5, Line 23: Missing word; "consolidated" should be lowercase.**
Thank you for identifying this error. We have corrected the sentence by adding the missing word "study" and changing "consolidated" to lowercase:<br>"The reporting of this qualitative study was guided by the consolidated criteria for reporting qualitative research (COREQ) 32-item checklist (see supplementary file 1) (18)."
**Page 5, Line 52: Wording unclear.**
Thank you - we have clarified the wording:<br>"We sought to purposively recruit consultants with a range of experiences, including those who had received performance pay, those who had applied unsuccessfully, and those who had not applied."
**Page 5, Line 60: Explain why only consultants with one year on site could be contacted.**

Only consultants who had been employed at the hospital site for at least one year were eligible for the award and were therefore eligible to participate in the study. We have clarified this in the manuscript as follows:<br>"Once the Trust had made decisions on performance pay awards, the hospital site administrator circulated information about the interview study to all consultants eligible for the award. Eligibility for the award required consultants to have been employed at the site for at least one year, and only these consultants were contacted."
**Page 7, Lines 8-11: The justification for TA over other methods is overstated. Other methods e.g. grounded theory, IPA, discourse analysis all involve interpretative analysis. I recommend revising the sentence to say TA is suitable or well-established.**
We agree and have reworded the sentence as follows:<br>"Thematic analysis was conducted by two research team members (ME and JP) to analyse the qualitative data, **as this approach is well suited to identifying patterns and themes across qualitative data (20)."**
**Page 7, Line 12: According the Braun and Clarke codes do not 'arise'. I suggest revising the wording. See Byrne, D. A worked example of Braun and Clarke's approach to reflexive thematic analysis. Qual Quant 56, 1391–1412 (2022).**
Many thanks - we have reviewed the article by Byrne, incorporated it into our manuscript and reworded as follows:<br>"Coding and analysis rarely operate in a purely inductive or deductive way; rather, most analyses involve a combination of both (29). In line with this, the qualitative data were analysed using both deductive and inductive approaches (i.e. applying codes derived from the topic guide as well as developing additional codes from the data) to ensure that the themes were grounded in and closely linked to the data (21,22)."
**Page 8, Some sub-themes appear misplaced or too broad - consider repositioning (see major comment).**
We have addressed this comment on the sub-themes and your concerns on the analysis outlined in the "major comment" section by refining the thematic structure, reviewing the positioning and scope of sub-themes, and ensuring that each sub-theme is clearly defined and distinct. We have also reduced the number of illustrative quotes and strengthened the accompanying interpretation to provide a more analytical and coherent presentation of the findings (see changes highlighted in the results section). As suggested by reviewer 2, we have now also provided a concept map of the study themes and subtheme (see Figure 2). Specifically, we have renamed one theme and three sub-themes to better reflect their content and scope (see Table 2).
**Page 8, Line 56: 'Given pandemic' needs rephrasing**.
We have rephrased it as follows:<br>"The appropriateness of this approach in the context of the coronavirus pandemic"
**Page 8, Equalisation' requires further explanation.**
Thank you we have reworded theme name to provide further explanation:<br>"Mixed views on the equal distribution of performance pay awards during the coronavirus pandemic"<br>We have updated the theme name throughout the article.
**Page 9, Line 55: Should read 'begging for the award.'**

Apologies. We have corrected this:
"These included the time it takes to complete the form, and negative connotations of feeling like they were asking or **begging for the award** as a result of the self-nomination element required."

Page 10
Line 38: Expand analysis so the quote is not doing all the work.
Line 49: Organisation culture seems quite broad here, this seems to be about misaligned incentives. If it's about culture I think this needs expanding and moving into a different section.

Comment on the text previously included on page 10, line 38:

Thank you we have expanded the analysis so the quote is not doing all the work: *"Despite positive reactions to the changes to the system and the application form, some limitations of the new system were reported. This included the separation of clinical and research elements, resulting in some applicants struggling to evidence research due to their clinical role."*

Comment on the text previously included on page 10, line 49:

Thank you - We have amended the sub-theme of 'Organisational Culture' to 'Misaligned priorities' to reflect the findings more accurately.

Page 11
Line 37: Missing space.

Apologies, we have corrected this:
*"... it's nice to have the money, but actually I think a lot of it is about, somebody has looked at what I've done and said, "We think that's valuable and we're grateful that you've done it." (***Consultant 11**, female)

Page 13
Lines 4-9: Quote needs clearer contextualisation (specialties, research time). Lines 12-35: Three similar quotes - consider reducing and strengthening analysis.
Lines 42-52: Expand on how the form is completed and how this disadvantages women returning from maternity leave.

Comment on text previously on page 13, 4-9:

Thank you, unfortunately, we cannot provide more context to this as no additional information was collected.

Comment on text previously on page 13, line 12-35:

Thank you we have revised this section and expanded on the analysis and reduced the number of quotes:

"Sole recognition rather than whole team
Some participants felt that performance pay awards were unfair as they recognise work of individuals whilst often the work has been conducted by the whole team. An individual award often felt unfair due to not recognising the contribution of the wider team.
"It doesn't take into account everybody else who facilitated me doing it. So, actually it should be a team recognition. Very rarely do, do, do we do things as absolute individuals although we may be the driving force." (Consultant 3, male)

"Cause they're assessing what you've done. So, I find that quite hard when it's not just me. And it nearly always isn't just me, 'cause we, we're teams. You know, we work in teams. And so, that's quite horrible, you know, having to kind of write yourself up." (Consultant 23, female)"

Comment on text previously on page 14 (rather than 13), line 42-52:

We believe that this comment referred to page 14, line 42-52 and have expanded on how the application system disadvantages women returning from maternity leave:
"Others emphasised that men's work is often valued more highly and that women face disadvantages related to maternity leave, putting them at a perceived detriment when looking back over achievements in the period covered by the application form, if some or all of that time has been spent on maternity leave, leaving them with less opportunity to demonstrate excellence. This reinforces systemic inequities in recognition and reward."

**Page 15**<br>**Line 37: Section contains quotes with minimal analysis - I suggest expanding the narrative.**<br>**Overall: Too many quotes in succession. I suggest developing the interpretive commentary.**

<u>Comment on previously page 15, line 37:</u><br>Thank you – we have expanded the narrative as follows:<br>"Other participants spoke less favourably about the temporary move towards equalisation during the pandemic. Some felt a sense of unfairness that even those staff that were unable to work during the pandemic were entitled to a share of the payment, with no recognition for those working above their normal roles."<br><u>Overall comment:</u><br>Here, and throughout the paper we have reduced the number of quotes, to include those that add value to the manuscript (please see results section). We have also tried to ensure that the explanation of the sub-themes are more detailed and so the quotes compliment/ illustrate the themes rather than relying on these for information about the theme.
**Page 16**<br>**Final theme also relies heavily on quotes – I suggest strengthening the analysis here and do not leave the quotes to do the heavy lifting.**
As above, we have expanded the explanation to explain the final theme better and removed some quotes.
**Page 17, Lines 44-46: I would have thought that non-clinical researchers in health research are fairly common is this commonly cited as a strength?**<br>**Many strengths listed are standard qualitative practice. I suggest focusing on what is distinctive.**
We thank the reviewer for this helpful comment. In response, we have revised the strengths and limitations section to focus on aspects of the study that are distinctive. We have removed the statement regarding non-clinical researchers conducting the interviews as a study strength, as well as other points that reflect standard qualitative research practice rather than specific strengths of this study:<br>"This was the first qualitative study to explore consultants' views and experiences on performance pay. The study adhered to the COREQ 32-item checklist, enhancing transparency and rigour in reporting qualitative research. Participants expressed both positive and negative views about the award process, including among those who had applied."
**Page 17, Line 58: Provide more detail on why unsuccessful applicants could not be recruited.**
We have provided more detail on why unsuccessful applicants could not be recruited:<br>"Recruitment emails and reminder emails explicitly emphasised our interest in hearing from consultants across all three groups (successful applicants, unsuccessful applicants, and non-applicants. However, due to the study timeline and funding constraints, the recruitment period was limited, and no consultant who had applied for an award unsuccessfully volunteered to participate. This limits the breadth of experiences captured."
**Page 18, Lines 33, 51, 57: Clarify that survey findings are from a separate publication and connect them more explicitly to the interview findings. I suggest stating the findings from the survey more clearly as in places it relies on the reader checking the other publication.**

Thank you for this suggestion. We have revised the manuscript to more clearly distinguish survey findings (reported in Gauly et al. (11)) from the interview findings in this paper, and to summarise the survey results more explicitly within the text. We have also strengthened the links between the two datasets to make clearer how the interview findings build on and extend the survey results:<br>Rewording of the text previously included on page 18, line 33:<br>**"Both our survey, reported separately (11),** and interviews indicate that consultants perceive the Clinical Excellence Award scheme as unfair to women. **In the survey, respondents reported perceptions of gender inequity in access to and success within the scheme (11), our interview data build on this by identifying mechanisms underpinning these perceptions, including women's lower likelihood of putting themselves forward due to systemic biases, reduced confidence, and societal**

**expectations. Consistent with wider literature, women's lower propensity to self-promote and apply for awards has been identified as a barrier in self-nomination-based schemes (25)".** (…)<br>Rewording of the text previously included on page 18, line 51:<br>**"Our survey (11) also found mixed views on the temporary equalisation of awards during the coronavirus (COVID-19) pandemic and whether this approach should continue. The survey highlighted polarised options, with some respondents viewing it as improving fairness and other questioning its appropriateness.** The interview findings provided further explanation for these divergent views, reflecting ongoing uncertainty about how equity should be operationalised within the scheme." (…) Rewording of the text previously included on page 18, line 57:<br>**"The survey (11) showed that some consultants chose not to apply due to tax implications and lack of time. Our interviews add nuance to this finding by suggesting that financial factors alone were not a strong motivator for engagement. Participants noted that potential award income could often be matched or exceeded more easily through additional shifts rather than through completing the application process, reinforcing the limited influence of financial incentives on participation.** This aligns with evidence that intrinsic motivations - such as professionalism and clinician- or patient-directed improvement strategies - may be more effective drivers of performance in healthcare than financial rewards (27)." (…)
**Reviewer: 2**<br>**Prof. Behdin Nowrouzi-Kia, University of Toronto**
**Comments to the Author:**<br>**Thank you for the opportunity to review your work. This study from the United Kingdom explores the National Health Service consultants perspective and lived-experience of the local clinical excellence award. Overall, it is a noteworthy study that provides insight on consultants views on the changes to performance-related pay in England. However, the paper has issues that must be addressed before it may be accepted for publication. I have structured my feedback for each section of the paper. I'd be happy to review the paper again, after the authors have had the opportunity to review, revise and resubmit their work.**
Many thanks for your time to review our study and for your detailed feedback. We have addressed all your comments below.
**Abstract**<br>**The authors should include the use of the COREQ checklist in this section. Moreover, please spell out the NHS in the abstract. Given the international audience of the journal, this is important to improve the readers understand of the context of the study.**

We have now included the use of the COREQ checklist into the method section of the abstract:<br>**"**Interviews were conducted online, audio-recorded, transcribed verbatim, and analysed thematically using both inductive and deductive coding. Data collection continued until thematic saturation was reached. The reporting of this qualitative study was guided by the consolidated criteria for reporting qualitative research (COREQ) 32-item checklist."<br>We have also spelt out the term NHS in the abstract to improve the readers' understanding of the context of the study:<br>"To explore National Health Service (NHS) consultants' views and experiences of the local Clinical Excellence Award (CEA) scheme following changes to performance-related pay in England."
**Introduction: This is a well written section that highlights the history of the NHS and the CEA. I'd like to see a stronger connection with the literature on the topic and the rationale for this study. The knowledge gap that the study is attempting to address needs to be further strengthened and articulated in the section. For the benefit of the reader, please provide a clear objective/aims/purpose statement.**

Thank you – we have strengthened the knowledge gap that the study is attempting to address and added the following text:<br><br>"Performance-related pay (PRP) is 'a compensation scheme in which an employee's pay is based partially or wholly on performance' (3)However, the use PRP in healthcare organisations remains "contentious and inconclusive" (4) (5). Concerns relate to use of public money, the potential inequity between staff working in team-based settings and the problematic nature of measuring performance (6–9). Furthermore, there has been limited attention on PRP in relation to doctors, and especially their views of such schemes. Whilst PRP can incentivise staff to work 'harder' (9), the impact of monetary rewards on work effort may be limited, when doctors' self-determination and intrinsic motivation is taken into account(10)."<br><br>We have also provided a clear study aim now in the introduction:<br>"The aim was to explore consultants' experiences and views of the newly introduced local performance pay awards system, including barriers to applying and experiences of the application process, and to examine how these experiences influenced their views on performance pay more generally."
**Methods: The authors have included the COREQ checklist. For the data analysis section, the authors need to expand the use of the thematic analysis, why they used both and deductive and inductive approach as well as an expanded discussion of the methodological rigor.**
We have now added justification on the use of thematic analysis and justified why both deductive and inductive approach was used. We also expanded the discussion of the methodological rigor:<br>"Thematic analysis was conducted by two research team members (ME and JP) to analyse the qualitative data, **as approach is well suited to identifying patterns and themes across qualitative data (20). Coding and analysis rarely operate in a purely inductive or deductive way; rather, most analyses involve a combination of both (29). In line with this, the qualitative data were analysed using both deductive and inductive approaches (i.e. applying codes derived from the topic guide as well as developing additional codes from the data) to ensure that the themes were grounded in and closely linked to the data (21,22). (….)"**<br>"Codes were discussed by the research team and themes reviewed and defined iteratively. **This collaborative and iterative process supported analytic rigour by enabling reflexive discussion of interpretations and ensuring consistency and coherence in coding and theme development across the dataset."**
**Results: It would be helpful if the authors also provided a concept map of the study themes and subtheme in additional to table 2. This will be a helpful visual to provide a high-yield overview of the study findings.**

Thank you for this helpful suggestion. We have created a concept map (Figure 1) illustrating the themes and sub-themes identified in the study, which is provided alongside Table 2 to offer a visual overview of the findings. We have also cited Figure 1 in the text as outlined below: “The themes and sub-themes identified in the analysis are presented in Table 2 and visually summarised in a concept map (Figure 1), providing an overview of the relationships between the key findings. The themes and sub-themes are described in detail below and are illustrated with direct quotations from participants.”
**Discussion**<br>**The structure of this section should be improved. I would suggest restating the purpose of the study, expanding on the results section using a critical lens and aligning the findings with the extant literature. I would also suggest expanding and delving into providing concrete recommendations for policy makers and employers. The limitation and conclusion sections should be expanded, including more details on future research consideration**
Thank you - we have now improved the structure of the dissertation. For example, we have revised some of the sub-headings (please see all changes in the discussion section). As suggested, we have restated the purpose of the study at the beginning of the discussion:<br><br>“The study focussed on consultants’ experiences and views of a newly introduced local performance pay awards system, including barriers to applying and experiences of the application process, and to examine how these experiences influenced their views on performance pay more generally.”<br><br>We have also expanded on the results section:<br><br>“In April 2024, consultants approved a new pay agreement (25), which incorporated Local Clinical Excellence Award (LCEA) budgets into basic pay (19). Nevertheless, the findings from this study offer important insights for the continued administration of National Clinical Impact Awards. More broadly, the research enhances our understanding of the benefits, limitations, and overall impact of performance-related pay within the healthcare sector.”<br><br>While we have not incorporated additional literature in the presentation of the key findings, we have expanded the discussion of findings in relation to the wider literature in the subsequent sections of the discussion:<br>“This concern is consistent with evidence from the UK Quality and Outcomes Framework, where financial incentives improved recorded quality of care in the short term but showed less consistent benefits over longer periods, with gains often reversing after incentives were withdrawn (41). Similarly, evaluation of the NHS Prescribed Specialised Services Commissioning for Quality and Innovation (PSS CQUIN) programme found that most pay-for-performance schemes had little or no measurable impact on quality improvements in specialised services, suggesting that incentives alone may be insufficient to drive sustained change when targets are complex or focused on specific measurable outcomes (42). A wider review across Organisation for Economic Co-operation and Development (OECD) countries also found mixed effects, with only just over half of studies demonstrating improved healthcare worker performance, emphasising the importance of carefully designed incentives that promote meaningful outcomes rather than narrow target achievement (43).”<br><br>“Ensuring that professionals understand how decisions are made and feel involved in the process is essential to translating financial recognition into genuine engagement rather than perceptions of unfairness or favouritism (44).”

As recommended, we have strengthened the section on recommendations for policy and practice by adding the following text to the relevant section:<br><br>“Policymakers and employers should therefore consider revising award criteria to place greater emphasis on sustained improvements in patient care, service development, education, research, and leadership over time, rather than isolated achievements.”<br><br>“With the 10-Year Health Plan for England proposing the expansion of financial rewards to high-performing teams, leaders, and organisations, policymakers should pilot and rigorously evaluate team-based reward models before wider implementation, assessing their effects on collaboration, staff morale, equity, and patient outcomes. Employers can support these initiatives by embedding team-based recognition within appraisal systems, ensuring that contributions from multidisciplinary teams are acknowledged alongside individual achievement.”
“Policymakers should therefore develop transparent but proportionate award processes, clearly communicating the criteria and rationale for awards while protecting individual financial confidentiality. Employers should complement these reforms by providing structured mentoring, application support, and leadership development opportunities for underrepresented groups, alongside regular equality audits to identify and address disparities in award applications and outcomes across gender, specialty, and other protected characteristics. Clearer and more inclusive definitions of excellence, supported by assessor training to minimise bias, may further improve confidence in the fairness of the process.”<br><br>“To maximise its effectiveness, policymakers should design reward systems that prioritise sustained impact, collaboration, and equity, while employers should ensure that implementation is transparent, inclusive, and supported by robust monitoring and evaluation. Together, these measures could help ensure that performance-related pay promotes meaningful improvements in workforce motivation, organisational culture, and ultimately, patient care.”<br><br>We have further expanded the ‘strength and weaknesses of the study’ section by adding the following text:<br><br>“Purposive sampling was used to recruit consultants with a range of experiences of the award process (successful applicants, unsuccessful applicants, and non-applicants), as well as a diversity of gender, ethnicity, and career stage. Recruitment and reminder emails explicitly emphasised our interest in hearing from all of these groups. However, all consultants who applied in this award round were successful, meaning there were no unsuccessful applicants available to participate. In addition, participation was voluntary, and recruitment was constrained by the study timeline and available funding. Consequently, the final sample was predominantly male, White British, and established consultants, potentially underrepresenting the perspectives of women, consultants from minority ethnic backgrounds, and less established consultants, who may be disproportionately affected by inequalities in the award process. This may have limited the breadth of perspectives and experiences captured.”

As recommended, we have expanded the conclusion to include more details on future research consideration:

"Future research should examine the long-term impact of evolving performance-related pay models, particularly team-based reward systems, on workforce motivation, collaboration, retention, and patient outcomes. Longitudinal and multi-site studies are also needed to evaluate whether policy changes reduce inequalities in award access and outcomes, and to identify strategies that support fairer, more inclusive recognition of excellence across the consultant workforce. Such evidence will be essential to inform the development of reward systems that not only recognise excellence but also foster sustained improvements in organisational performance and patient care."

VERSION 2 - REVIEW

**Reviewer**	**1**
**Name**	**Stringer, Gemma**
**Affiliation**	**University of Manchester, Division of Population Health**
**Date**	**03-Aug-2026**
**COI**	

Major comments

The authors have done a good job in addressing the comments and the paper has improved. I think there is still some work to do in organising the themes which are logical but would benefit from further refinement. It would also be good to consider using a more consistent style for theme titles and ensuring themes and subthemes are at a similar level of abstraction. The current themes are largely descriptive and there is some overlap between concepts such as transparency, anonymity and fairness. Braun and Clarke's approach to reflexive thematic analysis explains that themes should represent patterns of shared meaning, organised around a central organising concept. It would be worth considering reorganising the codes into a smaller number of more conceptual themes that represent the underlying meaning of the participants views e.g. experiences of the application process, motivations for engagement with the scheme, perceptions of fairness and equity, and the influence of organisational and contextual factors (including COVID) OR: experiences of the application process (e.g. structure, excellence, visibility, barriers, motivation); perceptions of fairness (e.g. speciality differences, team vs individual, gaming, female consultants, transparency/anonymity, COVID); organisational culture and recognition (e.g. recognition vs financial reward, discussing money, organisational priorities, organisational recognition).

I think there is a need for further restructuring of the discussion section. The discussion still relies heavily on the comparisons with the survey, whereas the focus should be on comparison with the wider literature. I suggest using the wider literature as the main structure and using the survey only when it provides a particularly relevant comparison or

adds value. One option would be to consolidate the survey comparisons into one clear part of this section or each paragraph could start with a discussion of the wider literature, followed by how the findings relate/extend/contrast with the survey. This would help to avoid starting paragraphs with reference to our survey or the survey, which currently makes the discussion feel as though it is framed around the survey rather than the wider evidence base. I would also suggest changing the last paragraph to focus on the interview findings rather than the combined findings. As its written this gives the impression that this paper includes the survey findings.

Minor comments:

Introduction

p.3. line 45 – should say "the use of"

p. 5 lines 35-44 – consider adding a line here to make it clear that consultants who chose not to apply participated e.g. after the line ending "…no unsuccessful applicants available to participate", add something like "Consultants who had chosen not to apply did participate in the study, alongside successful applicants."

Methods

p. 6. line 48 – should say "those who had not"

p.7 line 52 – I've not heard of "electronic face sheet" consider using a different term e.g. electronic form or digital form

p.7 line 53/54 – this could just say: "Participants could choose "prefer not to say" for any item."

p.8 line 11 – should say "this approach..."

Results

p.9 line 45 – the 5th theme is missing from the list

VERSION 2 - AUTHOR RESPONSE

**Reviewer 1 :**
**Major comments:**
**The authors have done a good job in addressing the comments and the paper has improved.**
Many thanks for your continued support in reviewing our manuscript; we greatly appreciate your time and constructive feedback. We hope that our latest revision satisfactorily addresses all of the comments raised. We have carefully revised the Results and Discussion sections and addressed all of the minor comments. We sincerely apologise for the errors in the previous version and can assure you that we have now given the entire manuscript another thorough proofread.

**I think there is still some work to do in organising the themes which are logical but would benefit from further refinement. It would also be good to consider using a more consistent style for theme titles and ensuring themes and subthemes are at a similar level of abstraction. The current themes are largely descriptive and there is some overlap between concepts such as transparency, anonymity and fairness. Braun and Clarke's approach to reflexive thematic analysis explains that themes should represent patterns of shared meaning, organised around a central organising concept. It would be worth considering reorganising the codes into a smaller number of more conceptual themes that represent the underlying meaning of the participants views e.g. experiences of the application process, motivations for engagement with the scheme, perceptions of fairness and equity, and the influence of organisational and contextual factors (including COVID) OR: experiences of the application process (e.g. structure, excellence, visibility, barriers, motivation); perceptions of fairness (e.g. speciality differences, team vs individual, gaming, female consultants, transparency/anonymity, COVID); organisational culture and recognition (e.g. recognition vs financial reward, discussing money, organisational priorities, organisational recognition).**
Thank you for this very helpful comment regarding the analysis and structure of the results. We have spent some more time reflecting on the results section, and have restructured this, in line with your suggestions. We have reduced the number of themes, and followed your suggestion, and the themes now consist of: Motivations for engagement with the scheme, Experience of the application process, Perceptions of fairness and equity and Influence of organisational and contextual factors.<br><br>We have also renamed many of the sub-themes. We hope this has improved the results section, and has also reduced some of the overlap mentioned. We have reflected the changes also in the abstract and in Figure 1 (concept map of themes and sub-themes).
**I think there is a need for further restructuring of the discussion section. The discussion still relies heavily on the comparisons with the survey, whereas the focus should be on comparison with the wider literature. I suggest using the wider literature as the main structure and using the survey only when it provides a particularly relevant comparison or adds value. One option would be to consolidate the survey comparisons into one clear part of this section or each paragraph could start with a discussion of the wider literature, followed by how the findings relate/extend/contrast with the survey. This would help to avoid starting paragraphs with reference to our survey or the survey, which currently makes the discussion feel as though it is framed around the survey rather than the wider evidence base. I would also suggest changing the last paragraph to focus on the interview findings rather than the combined findings. As its written this gives the impression that this paper includes the survey findings.**

Many thanks. We felt that it was important to retain comparisons with the survey where these provide useful context for the interview findings, but agree that the previous structure placed too much emphasis on the survey rather than the wider evidence base. We initially considered consolidating the survey comparisons into one part of the section but felt that this did not flow well. We have therefore followed your suggestion and restructured the section so that each paragraph begins with a discussion of the wider literature, followed by comparison with the survey only where this provides a relevant comparison or adds value. We have also added more relevant literature to strengthen the discussion. Finally, we have also revised the final paragraph to focus specifically on the interview findings, as suggested, to make clear that the survey findings are reported separately.
**Minor Comments:**
**Introduction: p.3. line 45 – should say "the use of"**
Apologies – we have corrected this error:<br>"However, the use **of** PRP in healthcare organisations remains "contentious and inconclusive" [4] [5]."
**Introduction: p. 5 lines 35-44 – consider adding a line here to make it clear that consultants who chose not to apply participated e.g. after the line ending "…no unsuccessful applicants available to participate", add something like "Consultants who had chosen not to apply did participate in the study, alongside successful applicants."**
Thank you – we have added a line here as suggested:<br>"However, in this award round, all consultants who applied were successful, meaning there were no unsuccessful applicants available to participate. **Consultants who chose not to apply did participate in the study, alongside the successful applicants.**"
**Methods: p. 6. line 48 – should say "those who had not"**
Apologies, we have corrected this:<br>"We sought to purposively recruit consultants with a range of experiences, including those who had received performance pay, those who had applied unsuccessfully, and those who **had** not applied."
**Methods: p.7 line 52 – I've not heard of "electronic face sheet" consider using a different term e.g. electronic form or digital form**
We have changed the wording as suggested:<br>"At the end of the interview, demographic information (years since qualification; gender; ethnicity; all in categories) was collected from participants using a digital form."
**Methods: p.7 line 53/54 – this could just say: "Participants could choose "prefer not to say" for any item."**
We have reworded the sentence as suggested:<br>"Participants could choose "prefer not to say" for any item."
**Methods: p.8 line 11 – should say "this approach..."**

Apologies – we have corrected this error:<br>"Thematic analysis was conducted by two research team members (ME and JP) to analyse the qualitative data, as **this approach** is well suited to identifying patterns and themes across qualitative data [28]."
**Results: p.G line 45 – the 5th theme is missing from the list**
Thank you for highlighting this. As we have restructured and renamed the themes in line with the point above, the 5th theme is no longer relevant. The themes are now listed as:<br>1. Motivations for engagement with the scheme, 2. Experience of the application process, 3. Perceptions of fairness and equity and 4. Influence of organisational and contextual factors.

VERSION 3 - REVIEW

**Reviewer**	**1**
**Name**	**Stringer, Gemma**
**Affiliation**	**University of Manchester, Division of Population Health**
**Date**	**01-Sep-2026**
**COI**	

The authors have engaged with the comments and the changes made have significantly improved the paper.

I suggest one small change, the first time the survey is mentioned in the discussion it should say "separately reported survey findings" at the moment this happens at the third mention of the survey.
