# Supplementary material for: National Health Service consultants’ experiences and views of performance pay in England: a qualitative interview study
Source: BMJ Open. 2026 Sep 28;16(9):e111699. doi: 10.1136/bmjopen-2025-111699 (PMC13636320; doi:10.1136/bmjopen-2025-111699)
Supplement: Supplementary file 2 [file bmjopen-16-9-s001.docx]

**Consolidated criteria for reporting qualitative studies (COREQ): 32-item checklist**

| No | Item | Guide questions/description | Page number |
| --- | --- | --- | --- |
| **Domain 1: Research team and reflexivity** | | | |
| ***Personal Characteristics*** |  |  |  |
| 1 | Interviewer/facilitator | Which author/s conducted the interview or focus group? | P.6 (Data collection/ Positionality) |
| 2 | Credentials | What were the researcher’s credentials? e.g. PhD, MD | P1. |
| 3 | Occupation | What was their occupation at the time of the study? | P.6 |
| 4 | Gender | Was the researcher male or female? | P.6 |
| 5 | Experience and training | What experience or training did the researcher have? | P.6 |
| ***Relationship with participants*** |  |  |  |
| 6 | Relationship established | Was a relationship established prior to study commencement? | P.6 |
| 7 | Participant knowledge of the interviewer | What did the participants know about the researcher? e.g. personal goals, reasons for doing the research | P.6 |
| 8 | Interviewer characteristics | What characteristics were reported about the interviewer/facilitator? e.g. bias, assumptions, reasons and interests in the research topic | P.6 |
| **Domain 2: Study design** | | | |
| ***Theoretical framework*** |  |  |  |
| 9 | Methodological orientation and theory | What methodological orientation was stated to underpin the study? e.g. grounded theory, discourse analysis, ethnography, phenomenology, content analysis | NA |
| ***Participant selection*** |  |  |  |
| 10 | Sampling | How were participants selected? e.g. purposive, convenience, consecutive, snowball | P.6 |
| 11 | Method of approach | How were participants approached? e.g. face-to-face, telephone, mail, email | P.6 |
| 12 | Sample size | How many participants were in the study? | P.7 |
| 13 | Non-participation | How many people refused to participate or dropped out? Reasons? | P.7 |
| ***Setting*** |  |  |  |
| 14 | Setting of data collection | Where was the data collected? e.g. home, clinic, workplace | P.5/6 |
| 15 | Presence of non-participants | Was anyone else present besides the participants and researchers? | NA |
| 16 | Description of sample | What are the important characteristics of the sample? e.g. demographic data, date | P.7/8 |
| ***Data collection*** |  |  |  |
| 17 | Interview guide | Were questions, prompts, guides provided by the authors? Was it pilot tested? | Supplementary File 1 Topic Guide |
| 18 | Repeat interviews | Were repeat interviews carried out? If yes, how many? | NA |
| 19 | Audio/visual recording | Did the research use audio or visual recording to collect the data? | P.7 |
| 20 | Field notes | Were field notes made during and/or after the interview or focus group? | NA |
| 21 | Duration | What was the duration of the interviews or focus group? | P.7 |
| 22 | Data saturation | Was data saturation discussed? | P.7 |
| 23 | Transcripts returned | Were transcripts returned to participants for comment and/or correction? | NA |
| ***Domain 3: Analysis and findings*** | | | |
| ***Data analysis*** |  |  |  |
| 24 | Number of data coders | How many data coders coded the data? | P.7 |
| 25 | Description of the coding tree | Did authors provide a description of the coding tree? | NA |
| 26 | Derivation of themes | Were themes identified in advance or derived from the data? | P.7 |
| 27 | Software | What software, if applicable, was used to manage the data? | NA |
| 28 | Participant checking | Did participants provide feedback on the findings? | NA |
| ***Reporting*** |  |  |  |
| 29 | Quotations presented | Were participant quotations presented to illustrate the themes/findings? Was each quotation identified? e.g. participant number | P.9-16 |
| 30 | Data and findings consistent | Was there consistency between the data presented and the findings? | P.8-16 |
| 31 | Clarity of major themes | Were major themes clearly presented in the findings? | P. 8-16 |
| 32 | Clarity of minor themes | Is there a description of diverse cases or discussion of minor themes? | P.8-16 |
