# Supplementary material for: National Health Service consultants’ experiences and views of performance pay in England: a qualitative interview study
Source: BMJ Open. 2026 Sep 28;16(9):e111699. doi: 10.1136/bmjopen-2025-111699 (PMC13636320; doi:10.1136/bmjopen-2025-111699)
Supplement: Supplementary file 3 [file bmjopen-16-9-s002.docx]

**TOPIC GUIDE FOR SEMI-STRUCTURED INTERVIEWS WITH NHS CONSULTANTS**

- Interviews are expected to take around 30-60 minutes.
- Introduction –The interviewer will introduce themselves and give a brief explanation of the study and the purpose of the interview to familiarise the participant with the aims and objectives of the study. The interview will reiterate that participation is voluntary, and they may withdraw at any time and that interview responses will be anonymized in any reporting or publication. Participants will have the opportunity to ask questions throughout the study. Verbal consent will be obtained and recorded and interview participants will be made aware of this.

1. **Can you tell me about your role at [TRUST NAME]?**

- **What is your specialty?**
- **How long have you been a consultant at [TRUST NAME]?**
- **Where did you work before?**

2. **Can you tell me what you think about performance pay for consultants in general?**
3. **What do you think about the current level of overall pay (basic + premia pay such as CEAs)?**
4. **Can you tell me when you first heard about performance pay for NHS consultants?**

- **By whom did you first hear about performance pay for NHS consultants?**
- **Where/In which context did you first hear about performance pay for NHS consultants?**

5. **Can you tell me why you decided to apply/not to apply for a performance pay award/ APR at [TRUST NAME]?**

- **Was your decision influenced by thoughts about:**
- **Income**
- **Recognition (by organization/peers)**
- **Deserving impact (patient outcomes, financial savings)**

6. **Can you tell me how you felt when you found out that you did/ did not receive an APR at [TRUST NAME]?**
7. **Can you tell me what you think about the application process for performance pay awards/ APR awards at [TRUST NAME]?**

- **Do the criteria reflect the ways in which your job should be assessed?**

8. **What are your thoughts about communicating who received an APR award at [TRUST NAME]?**

- **Should CEA awards be made public?**
- **Should CEA awards be made public to other doctors/[TRUST NAME] staff/ to the public?**
- **Should the value (amount of money) gained through APR awards be communicated (If yes, why and to whom; if no: why not?)**

9. **What impact do you think applying for and (not) receiving an APR award have on you?**

- **Does it impact your motivation/ sense of worth/ job satisfaction/morale?**
- **In your view, do you think your view is similar to other consultants at [TRUST NAME]?**

10. **How does the APR relate to other assessment (e.g., appraisal/revalidation/public reporting)?**
11. **What did you think about equally distributing the money for performance pay (as it was done during the COVID-19 pandemic?)**

**What are your thoughts on applying performance pay to other (clinical and non-clinical staff?)**

12. **Do you have any thoughts on performance pay should be provided in the future?**

- **What are your thoughts on clinical excellence awards being provided for several years**
- **When do you think consultants who received an award/did not receive an award should be allowed to apply again?**

**End Interview – Record responses for demographic information via face sheet**

***Signposting of participants for mental health support (where appropriate):***

- *Make participants aware that they can contact their GP for mental health support (https://www.mentalhealthatwork.org.uk)*
- *NHS Moodzone: Whatever you need to know about coping with stress, anxiety or depression, or just the normal emotional ups and downs of life (https://www.nhs.uk/conditions/stress-anxiety-depression)*
- *Samaritans: Confidential support for people experiencing feelings of distress or despair (www.samaritans.org.uk)*
- *Mental Health Foundation: Provides information and support for anyone with mental health problems or learning disabilities (www.mentalhealth.org.uk)*
- *Rethink Mental Illness: Support and advice for people living with mental illness (Help Line No: 0300 5000 927)*

**Invite any final questions and thank the participant for their time.**
